# Efficacy of Electrostimulation in Treating Major Depressive Disorder and Generalized Anxiety Disorder: A Literature Review

**DOI:** 10.1192/j.eurpsy.2025.2129

**Published:** 2025-08-26

**Authors:** J. R. Pinto Nasr, F. H. de Oliveira Bezerra, V. A. Masson, L. R. Ambrósio Filgueiras

**Affiliations:** 1 Universidade Paulista - Campus Campinas, Campinas, Brazil

## Abstract

**Introduction:**

Major Depressive Disorder (MDD) and Generalized Anxiety Disorder (GAD) are prevalent conditions that significantly affect quality of life. Many patients with MDD and GAD do not respond adequately to conventional therapies, such as psychotherapy and antidepressants, highlighting the need for alternative treatments. In this context, electrostimulation, particularly Transcranial Magnetic Stimulation (TMS) and Transcranial Direct Current Stimulation (tDCS), has shown promise by modulating brain activity to relieve symptoms.

**Objectives:**

To assess the efficacy of TMS and tDCS in treating MDD and GAD.

**Methods:**

This systematic literature review was conducted in SciELO, PubMed, covering the period from 2014 to 2024. After an initial selection of 15 articles, six studies were chosen based on relevance and reliability. The analysis focused on outcomes from controlled and randomized studies as well as systematic reviews and meta-analyses, involving TMS and tDCS.

**Results:**

The reviewed studies demonstrated that TMS and tDCS significantly reduce symptoms in MDD and GAD compared to placebo. One study evaluated the combined effect of tDCS with antidepressants, indicating a more pronounced clinical response and suggesting potential synergy between neuromodulation and pharmacotherapy in cortico-limbic circuits. However, larger sample sizes are needed to achieve robust statistical validation, clarifying the isolated impact of each modality and their combination.

**Conclusions:**

Both TMS and tDCS represent effective therapeutic alternatives for patients with MDD and GAD, especially those refractory to conventional approaches. Although promising, implementing these techniques faces challenges, including high costs, the need for specialized professionals, and stronger scientific validation to enable widespread use. Expanding clinical knowledge and disseminating evidence-based guidelines can promote safe access to and usage of these therapies.

**Disclosure of Interest:**

None Declared

